# Patient-Provider Communication and Colorectal Cancer Screening Completion Using Multi-target Stool DNA Testing

**DOI:** 10.1007/s13187-024-02479-w

**Published:** 2024-07-20

**Authors:** Xuan Zhu, Linda Squiers, Gabriel Madson, Leah Helmueller, Brian G. Southwell, Shama Alam, Lila J. Finney Rutten

**Affiliations:** 1 Robert D. and Patricia E. Kern Center for the Science of Healthcare Delivery, Mayo Clinic, 200 First Street SW, Rochester, MN 55905 USA; 2 RTI International, Research Triangle Park, NC USA; 3 Exact Sciences Corporation, Madison, WI USA; 4 Department of Quantitative Health Sciences, Mayo Clinic, Rochester, MN USA

**Keywords:** Colorectal cancer screening, Provider communication, Provider recommendation, Cancer education, Multi-target stool DNA (mt-sDNA)

## Abstract

Colorectal cancer (CRC) screening continues to be underutilized in the USA despite the availability of multiple effective, guideline-recommended screening options. Provider recommendation has been consistently shown to improve screening completion. Understanding how patient-provider communication influences CRC screening can inform interventions to improve screening completion. We developed a behavioral theory-informed survey to identify patient-provider communication factors associated with multi-target stool DNA (mt-sDNA) screening completion. The survey was administered by RTI International between 03/2022 and 06/2022 to a sample of US adults ages 45–75 who received a valid order for mt-sDNA screening with a shipping date between 5/2021 and 9/2021. Respondents completed an electronic or paper survey. Multivariable logistic regression was used to identify patient-provider communication factors associated with mt-sDNA test completion. A total of 2973 participants completed the survey (response rate, 21.7%) and 81.6% of them (*n* = 2427) reported having had a conversation with provider about mt-sDNA testing before the test was ordered. Having a conversation with the provider about the test, including discussions about costs, the need for follow-up testing and test instructions were associated with higher odds of test completion and being “very likely” to use the test in the future. Lack of discussion about advantages and disadvantages of available CRC screening options and lack of patient involvement in CRC screening decision-making were associated with reduced odds of test completion and likelihood of future use. Healthcare providers play a key role in patient adherence to CRC screening and must be appropriately prepared and resourced to educate and to engage patients in shared decision-making about CRC screening.

## Background

Colorectal cancer (CRC) is the third leading cause of cancer death in the United States (US) among both women and men [1, 2]. Several CRC screening tests, shown to reduce CRC incidence and mortality rates through early detection and removal of precancerous lesions, are recommended by major guideline organizations for adults between the ages of 45 and 75 years at average risk for CRC (i.e., no prior diagnosis of CRC, adenomatous polyps, or inflammatory bowel disease; no personal diagnosis or family history of known genetic disorders that predispose them to a high lifetime risk of CRC) [3–5]. The guideline-recommended CRC screening methods include stool-based modalities such as fecal immunochemical test/guaiac-based fecal occult blood test (FIT/gFOBT) annually and multi-target stool DNA (mt-sDNA) test every 3 years as well as direct visualization modalities such as screening colonoscopy every 10 years and computed tomography colonography and flexible sigmoidoscopy every 5 years [3, 5]. However, CRC screening continues to be underutilized in the US, particularly among uninsured and underinsured individuals, socioeconomically disadvantaged communities, and racial/ethnic minority populations [6–9].

Earlier studies have identified various factors at the patient, provider, and system levels that facilitate or impede CRC screening completion, with provider recommendation consistently reported to be a significant driver for completing CRC screening among individuals at average risk [10–14]. However, the communication between patients and providers concerning CRC screening is more complex than solely receiving a recommendation from the provider. Apart from the provider recommendation itself, this conversation may encompass discussions about the benefits and risks of screening, costs, procedures and preparation instructions, and patient preferences and concerns. Currently, there is limited data on how specific elements of patient-provider communication influence CRC screening completion [13, 15–18].

Gaining insight into the impact of different facets of patient-provider communication on CRC screening decision-making is crucial for developing effective health system- and provider-level interventions to improve CRC screening completion. This knowledge helps identify intervention targets and strategies that are likely to improve CRC screening completion. For instance, it can inform training and education efforts for providers, highlighting the specific information they should prioritize during CRC screening consultations. It can also inform the content of automated patient outreach communications to be implemented at the health system level. To address this knowledge gap, this study aims to identify patient-provider communication factors associated with average-risk patients’ completion of mt-sDNA testing (commercialized as Cologuard®) for CRC screening and their intention to complete mt-sDNA test in the future, using data from a health behavior theory-informed survey.

## Methods

### Survey Development

We developed a behavior theory-informed survey to examine the influence of psychosocial, communication, socio-demographic, and healthcare factors on average-risk patients’ completion of mt-sDNA test (Cologuard) and their intention to complete the test in the future. We iteratively revised the questionnaire based on findings from cognitive interviewing [19] with eight patients from the target population and an expert review using a systematic questionnaire appraisal process [20].

### Measures

Patient-provider communication factors were assessed using self-report measures. The measures were adapted from the Health Information National Trends Survey [21] or developed by the authors based on findings from Exact Sciences Corporation’s Cologuard patient experiences research. Respondents were asked whether they had a conversation with a healthcare provider about the mt-sDNA test before the test was ordered, followed by questions about contents of the conversation, including whether the provider discussed the cost of the test, the need to get a follow-up colonoscopy if they receive a result which detected potential signs of pre-cancerous growths or CRC, the cost of getting follow-up colonoscopy, the performance of the mt-sDNA test compared to other CRC screening tests, a basic overview of how to complete the test, and step-by-step instructions for completing the test. Respondents were also asked whether their provider described or recommended any other CRC screening tests including colonoscopy and FOBT/FIT. Additionally, they were asked how much their provider explained the advantages and disadvantages of available CRC screening tests and involved them in the decision about which test to get, using a 3-point scale (Not at all, Some, A great deal). Intention to use mt-sDNA test was measured by respondents’ reported likelihood of completing the mt-sDNA test in the future if their provider recommended it, on a 5-point scale (Not at all likely to Very likely). Respondents also self-reported sociodemographic and healthcare characteristics including age, sex, race, ethnicity, education level, employment status, marital status, health literacy, perceived adequacy of income, health insurance coverage, and last healthcare visit.

### Study Population

Eligible participants of this study were individuals with a US postal address who were between ages 45 and 75 and have had a mt-sDNA test kit ordered by their healthcare provider and shipped to them between May and September 2021. The final sampling frame included 17,370 individuals.

### Data Collection

RTI International staff conducted data collection between March and June 2022. We sent eligible participants an invitation letter by email if we had their email address (41.5%) or by postal mail. The invitation letter included an overview of the study and instructions to access the web survey. We sent a postcard reminder 1 week after the initial invitation, a letter and paper survey to non-respondents about 3 weeks after the initial invitation, and a final postcard reminder about 1 month after the initial invitation. A $20 incentive was provided to patients who completed the survey. --- Institutional Review Board reviewed the study and determined it exempt.

### Statistical Analysis

Descriptive statistics and chi-square test or Fisher’s exact test were used to summarize respondent reported provider-patient communication about CRC screening and examine differences in patient-provider communication by respondents’ mt-sDNA test completion status and by their intention to used mt-sDNA test in the future. Multivariable logistic regression was used to examine the associations between patient-provider communication about CRC screening and respondents’ mt-sDNA test completion status and the associations between patient-provider communication about CRC screening and respondents’ intention to use mt-sDNA test in the future, adjusting for respondents’ demographics and healthcare characteristics including age, sex, race, ethnicity, education level, employment status, marital status, health literacy, perceived adequacy of income, health insurance coverage, and healthcare visit in the last year. All analyses were conducted in R (Version 4.2.1) [22]. *p*-values < 0.05 were considered statistically significant.

## Results

A total of 2973 respondents completed the survey (response rate, 21.7%). We conducted a non-response analysis to examine whether there were differences between respondents (*n* = 2973) and non-respondents (*n* = 14,397) in sex and age. No significant differences in age or sex between respondents and non-respondents were observed. Table 1 summarizes participant demographics and healthcare characteristics. The majority was between 65 and 75 years of age (50.2%), female (66%), non-Hispanic (95.7%), White (88%), had at least some college education (77.1%), reported living comfortably on their present income (53.3%), and reported high health literacy (61.6%). All respondents had either private (59.7%) or public (40.3%) health insurance coverage.

**Table 1:** Participant characteristics by provider communication status

	Total (*N* = 2973)	Had a conversation with provider about the mt-sDNA test before they ordered it^a^		*p-*value^b^
		Yes (*n* = 2427)	No/don’t remember (*n* = 546)	
	*N* (%)	*N* (%)	*N* (%)	
Age in years				< .001
45–54	437 (14.70)	383 (15.78)	54 (9.89)	
55–64	1,045 (35.15)	875 (36.05)	170 (31.14)	
65 and older	1491 (50.15)	1169 (48.17)	322 (58.97)	
Sex				0.311
Male	1012 (34.04)	816 (33.62)	196 (35.90)	
Female	1961 (65.96)	1611 (66.38)	350 (64.10)	
Race^c^				
White	2615 (87.96)	2133 (87.89)	482 (88.28)	0.799
Black or African American	197 (6.63)	160 (6.59)	37 (6.78)	0.876
American Indian or Alaskan Native	37 (1.24)	29 (1.19)	8 (1.47)	0.668
Asian	91 (3.06)	81 (3.34)	10 (1.83)	0.073
Native Hawaiian or Other Pacific Islander	6 (0.20)	5 (0.21)	1 (0.18)	> 0.999
Other race	46 (1.55)	39 (1.61)	7 (1.28)	0.703
Ethnicity				0.221
Hispanic	126 (4.32)	98 (4.10)	28 (5.29)	
Non-Hispanic	2794 (95.68)	2293 (95.9)	501 (94.71)	
Education level				< 0.001
High school or less	665 (22.92)	503 (21.14)	162 (30.98)	
Some college	979 (33.74)	796 (33.46)	183 (34.99)	
Bachelor’s degree or more	1258 (43.35)	1080 (45.40)	178 (34.03)	
Employment status				< 0.001
Employed	1302 (44.15)	1134 (47)	168 (31.34)	
Retired/unemployed	1647 (55.85)	1279 (53)	368 (68.66)	
Marital status				< 0.001
Married	1924 (65.09)	1609 (66.63)	315 (58.23)	
Never married/separated/divorced/widowed	1032 (34.91)	806 (33.37)	226 (41.77)	
Health literacy				< 0.001
Extremely confident	1822 (61.53)	1530 (63.17)	292 (54.17)	
Very confident	761 (25.70)	600 (24.77)	161 (29.87)	
Somewhat confident	305 (10.30)	240 (9.91)	65 (12.06)	
Not at all/a little bit confident	73 (2.47)	52 (2.15)	21 (3.90)	
Perceived adequacy of income				< 0.001
Finding it difficult	324 (11.28)	247 (10.48)	77 (14.89)	
Getting by	1017 (35.40)	802 (34.04)	215 (41.59)	
Living comfortably	1532 (53.32)	1307 (55.48)	225 (43.52)	
Health insurance coverage				< 0.001
Public	1196 (40.30)	908 (37.47)	288 (52.84)	
Private	1772 (59.70)	1515 (62.53)	257 (47.16)	
Last healthcare visit				< 0.001
Less than a year ago	2610 (88.29)	2163 (89.53)	447 (82.78)	
At least a year ago or longer	346 (11.71)	253 (10.47)	93 (17.22)	

^a^*mt-sDNA* multi-target stool DNA

^b^*p*-values obtained from chi-square test or Fisher’s exact test

^c^Percentages may add up to over 100% because some participants selected multiple races

Table 2 summarizes differences in patient-provider communication about CRC screening by completers and non-completers of mt-sDNA test and between respondents who said they were “very likely” to complete mt-sDNA test in the future and respondents who were not. Of all respondents, 81.6% (*n* = 2427) reported that they had a conversation with their healthcare provider about the mt-sDNA test before the test was ordered for them. Among those who had a conversation with a healthcare provider about mt-sDNA test, statistically significantly higher proportions of completers (versus non-completers) and higher proportions of respondents who said they were “very likely” to complete mt-sDNA test in the future (versus respondents who were not) reported that their provider explained: the cost of mt-sDNA test (45.5% vs. 31.9%, 45.5% vs. 32.8%); the need for colonoscopy follow-up (82.2% vs. 64.1%, 81.8% vs. 66.7%); cost of follow-up colonoscopy (26.7% vs. 20.1%, 26.4% vs. 21.4%); performance of mt-sDNA test compared to other CRC screening tests (60.4% vs. 45.1%, 60.9% vs. 42.6%); overview of mt-sDNA test (63.1% vs. 43.7%, 62.6% vs. 46.3%); and step-by-step instructions to use the test (27.1% vs. 14.1%, 27.1% vs. 14.6%). Additionally, statistically significantly higher proportions of completers (versus non-completers) and higher proportions of respondents who said that they were “very likely” to complete mt-sDNA test in the future (versus respondents who were not) reported that their provider described alternative screening methods including colonoscopy (68.6% vs. 58.1%, 68.7% vs. 57.6%) and FOBT/FIT (19.5% vs. 13.4%, 19.3% vs. 13.9%). These respondents were also statistically significantly more likely to report “a great deal” (versus “some” or “not at all”) when asked how much their provider explained the advantages and disadvantages of CRC test (27.86% vs. 16.87%, 28.6% vs. 14.1%) and how much their provider involved them in decision about which screening test to get (54.5% vs. 34.0%, 55.8% vs. 29.1%).

**Table 2:** Patient-provider communication about colorectal cancer screening and mt-sDNA test (Cologuard) completion^a^

	Total (*N* = 2973)	Completers (*n* = 2299)	Non-completers (*n* = 674)	*p*-value^c^	“Very likely” to use mt-sDNA test in future (*n* = 2282)	Not “Very likely” to use mt-sDNA test in future (*n* = 659)	*p*-value^c^
	*N* (%)^b^	*N* (%)^b^	*N* (%)^b^		*N* (%)^b^	*N* (%)^b^	
Had a conversation with provider about the mt-sDNA test before they ordered it	2427 (81.63)	1979 (86.08)	448 (66.47)	< 0.001	1942 (85.10)	465 (70.56)	< 0.001
In the conversation about mt-sDNA test, the provider explained:^d^							
Cost of mt-sDNA test	1035 (43.04)	894 (45.54)	141 (31.90)	< 0.001	875 (45.45)	151 (32.75)	< 0.001
Need for colonoscopy follow-up	1889 (78.87)	1607 (82.20)	282 (64.09)	< 0.001	1568 (81.84)	307 (66.74)	< 0.001
Cost of follow-up colonoscopy	612 (25.49)	524 (26.69)	88 (20.09)	0.004	507 (26.37)	98 (21.35)	0.027
Performance of mt-sDNA test compared to other tests	1381 (57.61)	1183 (60.42)	198 (45.10)	< 0.001	1170 (60.94)	195 (42.58)	< 0.001
Basic overview of how to complete mt-sDNA test	1433 (59.51)	1240 (63.07)	193 (43.67)	< 0.001	1207 (62.57)	213 (46.30)	< 0.001
Step-by-step instruction of mt-sDNA test	593 (24.70)	531 (27.06)	62 (14.12)	< 0.001	521 (27.08)	67 (14.63)	< 0.001
Before ordering mt-sDNA test, provider described:							
Colonoscopy	1944 (66.24)	1560 (68.60)	384 (58.09)	< 0.001	1547 (68.66)	376 (57.58)	< 0.001
FOBT/FIT	524 (18.09)	437 (19.45)	87 (13.41)	< 0.001	429 (19.25)	89 (13.91)	0.002
Besides mt-sDNA test, provider recommended:							
Colonoscopy	1316 (44.70)	1026 (44.98)	290 (43.74)	0.572	1008 (44.54)	297 (45.48)	0.671
FOBT/FIT	245 (8.49)	203 (9.03)	42 (6.57)	0.049	187 (8.39)	54 (8.57)	0.885
Provider explained advantages and disadvantages of CRC tests:				< 0.001			< 0.001
A great deal	751 (25.39)	639 (27.86)	112 (16.87)		650 (28.57)	92 (14.05)	
Some	1716 (58.01)	1335 (58.20)	381 (57.38)		1296 (56.97)	403 (61.53)	
Not at all	491 (16.60)	320 (13.95)	171 (25.75)		329 (14.46)	160 (24.43)	
Provider involved respondent in decision about which screening test to get:				< 0.001			< 0.001
A great deal	1466 (49.85)	1240 (54.46)	226 (34.04)		1262 (55.84)	190 (29.10)	
Some	982 (33.39)	724 (31.80)	258 (38.86)		672 (29.73)	300 (45.94)	
Not at all	493 (16.76)	313 (13.75)	180 (27.11)		326 (14.42)	163 (24.96)	

^a^*mt-sDNA* multi-target stool DNA

^b^Not all *N*s add to total sample size due to missing responses; missing responses range from 5 to 41

^c^*p*-values obtained from chi-square test

^d^Only include respondents who reported have had a conversation with their provider about mt-sDNA test before the test was ordered

Table 3 summarizes the associations between patient-provider communication about CRC screening and mt-sDNA test completion and intention to complete mt-sDNA test in the future, adjusted for respondent sociodemographic and healthcare characteristics. The results showed that having a conversation with their provider about mt-sDNA test before the test kit was ordered was associated with higher odds of respondents completing mt-sDNA testing (OR = 2.23; 95%CI 1.74, 2.85) and being “very likely” to use mt-sDNA test in the future (OR = 1.60; 95%CI 1.24, 2.06). Feeling their provider did not explain the advantages and disadvantages of available CRC tests at all (versus explained “a great deal”) (OR = 0.67; 95%CI 0.47, 0.97) and feeling their provider allowed them only some involvement (versus “a great deal”) (OR = 0.59; 95%CI 0.47, 0.75) or no involvement at all (OR = 0.54; 95%CI 0.40, 0.74) in the decision about which screening test to get was associated with lower odds of respondents completing mt-sDNA testing. Additionally, their provider recommending colonoscopy in addition to mt-sDNA test (OR = 0.65; 95%CI 0.52, 0.82), feeling their provider explained the advantages and disadvantages of available CRC tests only to “some” extent (OR = 0.61; 95%CI 0.45, 0.81) or “not at all” (versus explained “a great deal”) (OR = 0.48; 95%CI 0.33, 0.70), and feeling their provider allowed them only some involvement (versus “a great deal”) (OR = 0.44; 95%CI 0.35, 0.56) or no involvement at all (OR = 0.49; 95%CI 0.35, 0.67) in the decision about which screening test to get was associated with lower odds of respondents reporting “very likely” to use mt-sDNA test in the future.

**Table 3:** Associations between provider-patient communication about colorectal cancer screening and mt-sDNA test (Cologuard) completion^a^^,b^

Associations between patient-provider communication factors and test completion		
	Model 1: Odds of completing mt-sDNA test	Model 2: Odds of being “very likely” to use mt-sDNA test in the future
	Odds ratio (95%CI)	Odds ratio (95%CI)
Had a conversation with provider about the mt-sDNA test before they ordered it	2.225 (1.737, 2.846)	1.598 (1.236, 2.062)
Before ordering mt-sDNA test, provider described:		
Colonoscopy	1.046 (0.823, 1.327)	1.214 (0.953, 1.544)
FOBT/FIT	1.018 (0.738, 1.420)	0.996 (0.716, 1.402)
Besides mt-sDNA test, provider recommended:		
Colonoscopy	0.808 (0.649, 1.005)	0.654 (0.523, 0.817)
FOBT/FIT	1.195 (0.772, 1.876)	0.806 (0.530, 1.234)
Provider explained advantages and disadvantages of available CRC tests:		
A great deal	Reference	Reference
Some	0.787 (0.598, 1.031)	0.609 (0.454, 0.811)
Not at all	0.671 (0.466, 0.967)	0.484 (0.331, 0.704)
Provider involved respondent in decision about which screening test to get		
A great deal	Reference	Reference
Some	0.593 (0.469, 0.749)	0.438 (0.345, 0.555)
Not at all	0.541 (0.396, 0.742)	0.486 (0.353, 0.671)
Associations between aspects of patient-provider communication about mt-sDNA test (Cologuard) and test completion ^c^		
	Model 3: Odds of completing mt-sDNA test	Model 4: Odds of being “very likely” to use mt-sDNA test in the future
	Odds ratio (95%CI)	Odds ratio (95%CI)
In the conversation about mt-sDNA test, the provider explained:		
Cost of mt-sDNA test	1.312 (1.007, 1.716)	1.304 (1.004, 1.699)
Need for colonoscopy follow-up	1.997 (1.522, 2.617)	1.573 (1.196, 2.064)
Cost of follow-up colonoscopy	0.729 (0.530, 1.007)	0.658 (0.484, 0.899)
Performance of mt-sDNA test compared to other tests	1.227 (0.952, 1.581)	1.561 (1.217, 2.004)
Basic overview of how to complete mt-sDNA test	1.479 (1.138, 1.926)	1.325 (1.024, 1.718)
Step-by-step instructions of mt-sDNA test	2.006 (1.395, 2.921)	1.832 (1.293, 2.623)

^a^*mt-sDNA* multi-target stool DNA

^b^All models adjusted for age, sex, race, ethnicity, education level, employment status, marital status, health literacy, perceived adequacy of income, health insurance coverage, and healthcare visit in last year

^c^Only include respondents who reported have had a conversation with their provider about mt-sDNA test before the test was ordered

Among those who had a conversation with their provider about mt-sDNA test, discussions with provider about the cost of mt-sDNA testing (OR = 1.31; 95%CI 1.01, 1.72), the need for a colonoscopy follow-up if receiving an abnormal result (OR = 2.00; 95%CI 1.52, 2.62), a basic overview of how to complete the mt-sDNA test (OR = 1.48; 95%CI 1.14, 1.93), and step-by-step instructions for completing the mt-sDNA test (OR = 2.00; 95%CI 1.40, 2.92) were associated with higher odds of respondents completing mt-sDNA testing. Similarly, discussions with their provider about the cost of mt-sDNA testing (OR = 1.30; 95%CI 1.00, 1.70), the need for a colonoscopy follow-up if received abnormal result (OR = 1.57; 95%CI 1.20, 2.06), performance of the mt-sDNA test compared to other CRC screening tests (OR = 1.56; 95%CI 1.22, 2.00), a basic overview of how to complete the mt-sDNA test (OR = 1.33; 95%CI 1.02, 1.72), and step-by-step instructions for completing the mt-sDNA test (OR = 1.83; 95%CI 1.29, 2.62) were associated with higher odds of respondents reporting “very likely” to use mt-sDNA test in the future. However, discussion with their provider about the cost of the follow-up colonoscopy was associated with lower odds of respondents reporting “very likely” to use mt-sDNA test in the future (OR = 0.66; 95%CI 0.48, 0.90).

## Discussion

Using survey data from a national sample of patients who were prescribed the test, our study examined the impact of patient-provider communication factors on completion of the mt-sDNA test and intention to use it for CRC screening in the future. A notable strength of our study is patients’ self-reports of the specific contents of the patient-provider communication. These data allowed us to identify key information that influences patients’ decision to complete CRC screening using the mt-sDNA test. Our findings suggest potential communication targets and strategies for interventions at system, provider, and patient levels to enhance CRC screening rates.

We found that having a conversation with the provider before ordering the mt-sDNA test, especially discussions about the cost, potential follow-up testing, and instructions on how to complete the test were associated with increased odds of test completion and being likely to use it in the future. Conversely, feeling that their provider did not adequately explain the advantages and disadvantages of available CRC screening tests, and having limited or no involvement in the decision-making process regarding the choice of screening test reduced the odds of test completion and intention to use it in the future. Discussions about costs of follow-up colonoscopy also reduced intention to use it in the future.

Our findings emphasize the importance of effective patient-provider communication in promoting CRC screening completion and future adherence. The provision of comprehensive information, including cost considerations, follow-up procedures, performance comparisons, and clear step-by-step instructions, appears to positively influence patients’ screening decisions. In contrast, limited information and the lack of involvement in decision-making hinder screening completion and adherence. Additionally, our finding underscores the significance of recent policy changes requiring CMS and private health insurers to cover the cost of follow-on colonoscopy and the need to communicate this policy to patients.

Health systems interested in improving CRC screening uptake should develop interventions aimed at improving patient-provider communication regarding CRC screening. For example, standardized protocols for CRC screening discussions could be implemented to support providers in communicating accurate and clear information about costs and follow-up procedures, discussion of essential steps of the screening process, and active involvement of patients in decision-making. Provider training may be needed to improve provider knowledge and skills on effective communication, including how to actively involve patients in shared decision making. For example, providers could use clinical decision and conversation aids to facilitate discussions about the advantages and disadvantages of available screening methods and how they align with the patient’s values, needs, and preferences, with the goal to help patients make informed decisions about screening that are well-suited to their individual circumstances [23–25]. Furthermore, health systems should consider interventions to increase patient access to comprehensive cancer screening information. Such interventions may include using telehealth visits for cancer screening consultations, designating cancer screening education responsibilities to nonphysician staff, implementing automated patient reminders and distributing easy-to-understand educational materials about cancer screening in multiple formats including brochures and online resources, and partnering with community organizations to reach underserved populations to promote cancer screening awareness and knowledge [26]. These interventions should ensure that educational materials are available in multiple languages and are culturally appropriate to diverse patient populations [26–28].

Our findings suggest a number of future research directions. First, future research could extend our study to identify the specific elements of patient-provider communication that effectively influence patient CRC screening decision-making and enhance screening completion, including examining how specific communication strategies affect patient choice and completion of different CRC screening tests. Second, future research could focus on developing and evaluating provider training programs designed to improve communication skills related to discussing CRC screening options, particularly regarding how to tailor communication to patient health literacy levels and cultural backgrounds. Moreover, future research could develop and test patient education materials, such as informational brochures and digital resources, to improve understanding of CRC screening tests and completion rates among patients from diverse sociodemographic groups.

This study is not without limitations. First, the cross-sectional survey design prohibits confirming causal relationships. Future research should use longitudinal designs to track screening behaviors over time and assess the long-term impact of patient-provider communication on CRC screening adherence. Second, reliance on self-reported data for measuring patient-provider communication may introduce recall bias, which can be addressed by combining patient self-reports with observer ratings of patient-provider interactions. Third, selection bias may have affected our findings. Although respondents and non-respondents did not differ by age and sex, there may have been additional unmeasured differences between these groups (e.g., health insurance, education) that may introduce bias. However, we were unable to compare these characteristics between participants and non-participants to assess potential biases. Additionally, survey respondents may be more likely to engage in conversations with others, including providers, which could influence the outcomes. Fourth, the limited representation of racial/ethnic minorities and lower SES participants restricted subgroup analysis and may limit generalization of our findings. Moreover, generalizing our findings to uninsured populations should be done with caution, given that all participants had health insurance coverage. Future research should consider oversampling underrepresented groups to inform targeted interventions tailored to specific community needs.

## Conclusions

Our study highlights the critical role of patient-provider communication in CRC screening completion and future likelihood of screening. The provision of information on costs, follow-up procedures, performance comparisons, and clear instructions as well as sufficiently involving patients in decision-making positively influences patients’ screening decisions. To improve CRC screening rates and promote cancer prevention, healthcare providers must be appropriately prepared and resourced to educate and engage patients in shared decision-making about CRC screening.

## Data Availability

The data that support the findings of this study are available from the corresponding author upon reasonable request.
